# Highly potent intracellular membrane-associated Aβ seeds

**DOI:** 10.1038/srep28125

**Published:** 2016-06-17

**Authors:** Anne-Marie Marzesco, Matthias Flötenmeyer, Anika Bühler, Ulrike Obermüller, Matthias Staufenbiel, Mathias Jucker, Frank Baumann

**Affiliations:** 1 Department of Cellular Neurology, Hertie Institute for Clinical Brain Research, University of Tübingen, D-72076 Tübingen, Germany; 2 German Center for Neurodegenerative Diseases (DZNE), D-72076 Tübingen, Germany; 3 Max Planck Institute for Developmental Biology, D-72076 Tübingen, Germany

## Abstract

An early event in Alzheimer’s disease (AD) pathogenesis is the formation of extracellular aggregates of amyloid-β peptide (Aβ), thought to be initiated by a prion-like seeding mechanism. However, the molecular nature and location of the Aβ seeds remain rather elusive. Active Aβ seeds are found in crude homogenates of amyloid-laden brains and in the soluble fraction thereof. To analyze the seeding activity of the pellet fraction, we have either separated or directly immunoisolated membranes from such homogenates. Here, we found considerable Aβ seeding activity associated with membranes in the absence of detectable amyloid fibrils. We also found that Aβ seeds on mitochondrial or associated membranes efficiently induced Aβ aggregation *in vitro* and seed β-amyloidosis *in vivo*. Aβ seeds at intracellular membranes may contribute to the spreading of Aβ aggregation along neuronal pathways and to the induction of intracellular pathologies downstream of Aβ.

Deposition of amyloid-β peptide (Aβ) in extracellular plaques is a major pathological hallmark of Alzheimer’s Disease (AD). Misfolding and subsequent aggregation of Aβ peptides are considered early steps in AD pathogenesis. However, although intensively studied, the mechanisms underlying the induction and spreading of the Aβ pathology in brain remain unclear, but are thought to include a prion-like seeding process^1^. *In vivo* experiments have demonstrated that β-amyloidosis can be induced in brains of young β-amyloid precursor protein (APP) transgenic mice by intracerebral injection of crude homogenates of aggregated Aβ-laden brains^2^,^3^.

Given that depletion of Aβ from such homogenates suppressed cerebral β-amyloid induction, the aggregates of misfolded Aβ have been postulated to be a major component of the Aβ seeding activity^2^,^4^. Consistently purified Aβ fibrils as well as aggregated synthetic Aβ preparations have been demonstrated to exhibit Aβ seeding activity although the later are much less efficient^5^,^6^. Follow-up studies revealed Aβ seeding activities in both the 100,000 × g PBS-soluble fraction and the pellet fraction which includes amyloid fibrils but also membrane vesicles^3^. Of note, Aβ has been located to various intracellular membranes^7^,^8^,^9^,^10^,^11^,^12^ but also to extracellular vesicles secreted from multivesicular bodies (MVBs.)^13^,^14^. Thus Aβ seeding activity in the pellet fraction could be associated with membranes which in turn have been implicated in misfolding and aggregation of Aβ^15^,^16^,^17^,^18^.

Here, we used subcellular fractionation to isolate membrane fractions from Aβ plaque bearing APP transgenic brain extracts. From both *in vitro* and *in vivo* assays our results demonstrate highly potent Aβ seeds associated with intracellular membranes including mitochondrial and associated membrane structures. Aβ seeds associated with intracellular membranes may contribute to the spreading of Aβ aggregation along neuronal pathways and to the induction of intracellular pathologies downstream of Aβ.

## Results and Discussion

### Differential distribution of *in vitro* Aβ seeding activity in crude brain fractions

**Figure 1:**
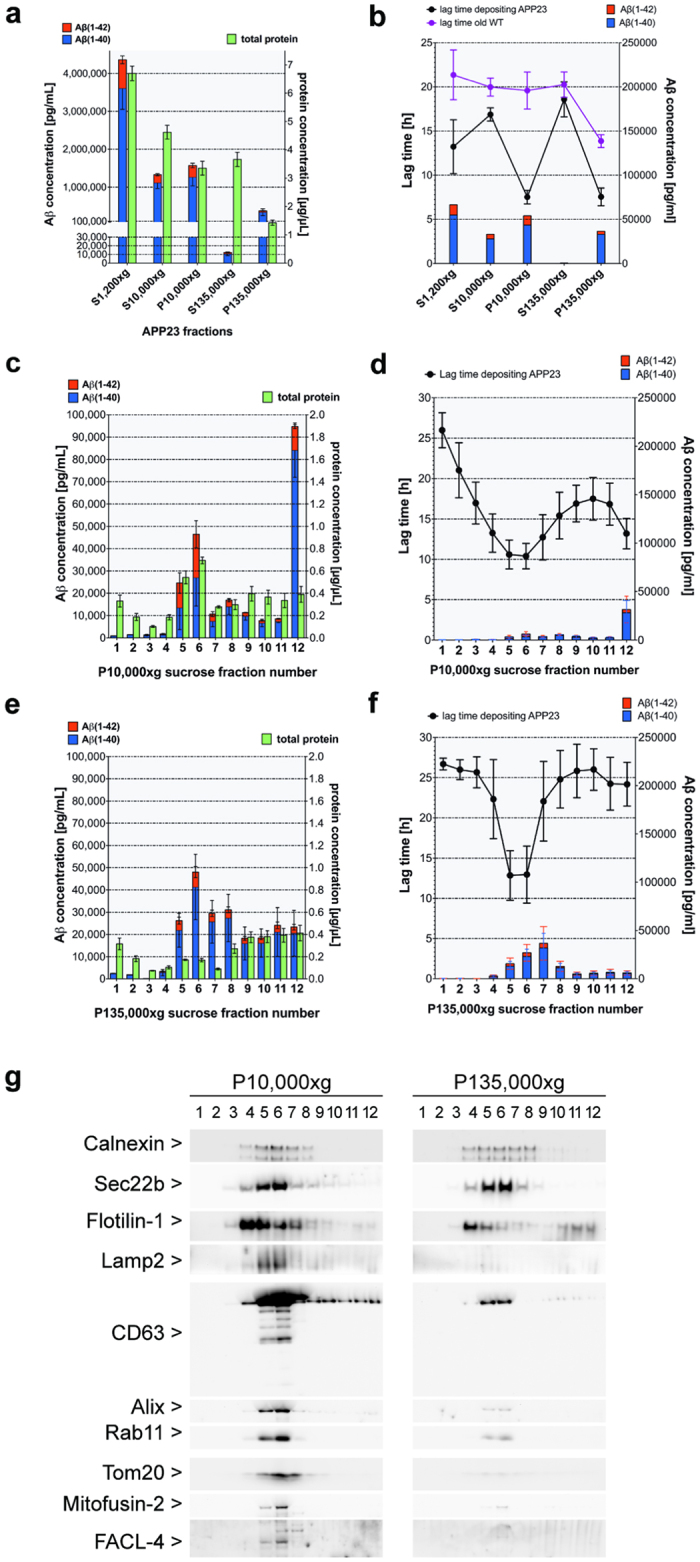
Fractionation of β-amyloid-laden APP23 transgenic brain and identification of *in vitro* seeding-active membrane-associated Aβ. (**a,b**) Supernatant (S) and membrane pellets (P) from brain after differential centrifugation at 1,200 × g (S1,200 × g), 10,000 × g (S10,000 × g and P10,000 × g) and 135,000 × g (S135,000 × g and P135,000 × g) (n = 6; always refers to independent fractionations from different brains) and (**c–f**) fractions from flotation sucrose density gradients of P10,000 × g (**c,d**) and P135,000 × g (n = 4) (**e,f**) membrane pellets. (**a,c,e**) Concentrations of total protein (green, right Y axis) and formic acid (FA) extracted Aβ(1–40) (blue) and Aβ(1–42) (red) (left Y axis) measured by ELISA. (**b,d,f**) Corresponding *in-vitro* seeding (FRANK) assays of (**b**) differential centrifugation fractions from APP23 and wild-type mice (n = 3), or of (**d,f**) sucrose gradient fractions from P10,000 × g (**d**) and P135,000 × g (**f**) of APP23 (n = 4). Note that the FRANK assay contains 0.1 μg total proteins for each fraction. The lag times for fibrillation of 25 μM soluble Aβ(1–40) are shown (black: APP23, purple: WT) together with the assay concentrations of Aβ(1–40) (blue) and Aβ(1–42) (red). Data are presented as mean ± SEM. (**g**) Western blots with equal volumes of P10,000 × g and P135,000 × g sucrose gradient fractions detecting marker proteins of endoplasmic reticulum (Calnexin, sec22b), of lipid rafts (Flotilin-1), of lysosomes/late endosomes (Lamp2), of exosomes (CD63, Alix, Rab11) and of mitochondria (Tom20), as well as of two specific mitochondrial-associated ER membranes (MAMs) markers Mitofusin-2 (MFN2) and Fatty acid CoA ligase 4 (FACL4). Note that full-length blots/gels are presented in Supplementary Fig. 6.

To assess the Aβ seeding activity at intracellular membranes we performed first subcellular fractionation and intracellular organelle purification based on established protocols (Supplementary Fig. 1) from aggregated Aβ-laden brain of aged APP23 mice^2^,^19^. After differential centrifugation (Supplementary Fig. 1) Aβ concentration was highest in the organelle-containing fractions (Supernatant (S) of 10,000 × g, Pellet (P) of 10,000 × g and P135,000 × g) (Fig. 1a). To evaluate *in vitro* the seeding activity of the subcellular fractions, we used the FRANK assay (Fibrillisation of Recombinant Aβ Nucleation Kinetic)^18^ which measures active Aβ seeds as they shorten the lag time until Aβ(1–40) monomers fibrilize (Supplementary Fig. 2). Normalized for total protein, we observed high Aβ seeding activity relative to the wild-type fractions (WT) in the resuspended P135,000 × g and even more in P10,000 × g (Fig. 1b). Notably, the seeding activities did not correlate with the respective Aβ amounts.

To further isolate and purify the intracellular membrane vesicles that bear the highest β-amyloid seeding activity, P10,000 × g and P135,000 × g were loaded on a flotation sucrose gradient (Supplementary Fig. 1). Total protein and Aβ concentrations differed in distribution along both sucrose gradients, with Aβ peaking in fractions 5/6/12 of the P10,000 × g gradient (Fig. 1c) and in fractions 5/6/7/8 of the P135,000 × g gradient (Fig. 1e). Non-membranous material was pelleted in fraction 12 of the P10,000 × g. Subsequently, the highest seeding activities were identified in fractions 5/6 of both P10,000 × g and P135,000 × g using the FRANK assay (Fig. 1d,f). Again, the seeding activities *in vitro* did not strictly correlate with the Aβ levels in the fractions (Fig. 1d,f) suggesting that only a subset of Aβ contributes to the seeding activity; which might be in a distinct more seeding active conformation.

### Highest Aβ seeding activity in mitochondria/MAM enriched fractions

**Figure 2:**
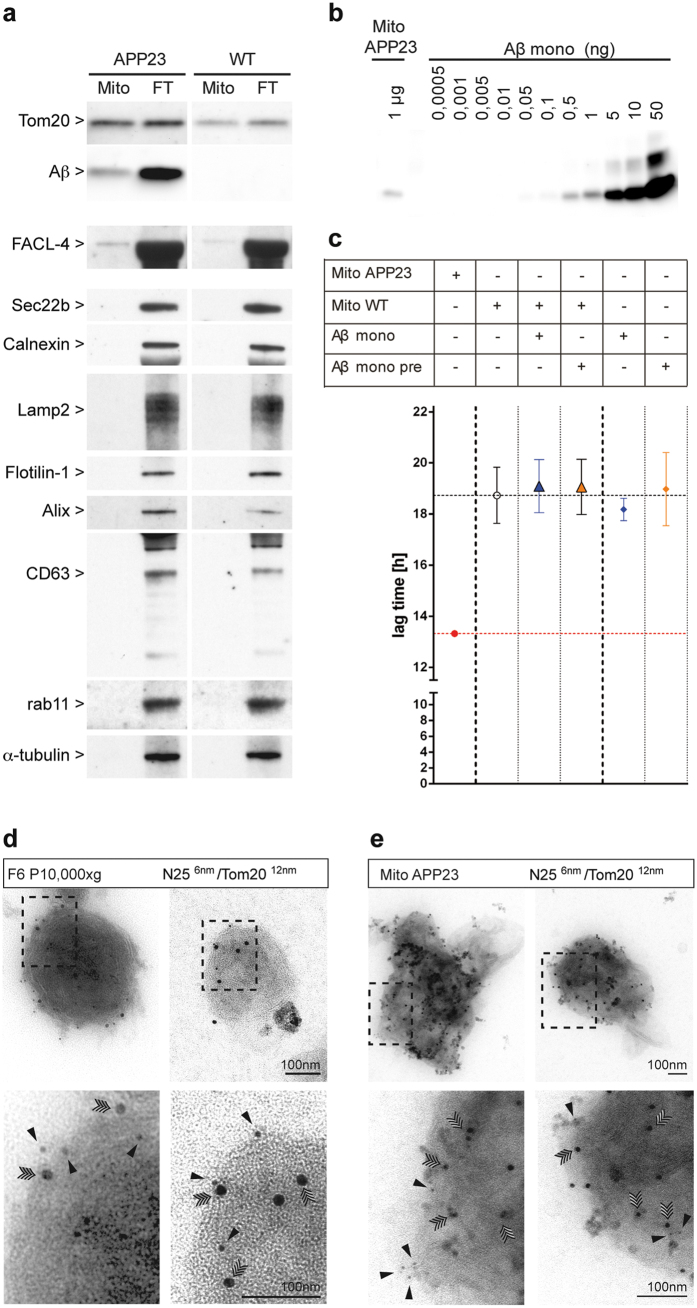
Immunoisolated mitochondria and associated ER-membranes contain Aβ and exhibit *in-vitro* seeding activity. (**a**) Immunoisolated mitochondria and the corresponding flowthrough were prepared from amyloid-laden transgenic APP23 (n = 3) and old wild-type C57BL/6 mice (n = 3) and analyzed by immunoblotting for Aβ peptide (monoclonal Aβ 6E10), and markers for mitochondria (Tom20 and FACL4) endoplasmic reticulum (sec22b, Calnexin), lysosomes/late endosomes (Lamp2), lipid rafts (Flotilin-1), exosomes (Alix, CD63 and Rab11) and cytoskeletal protein α-tubulin. Note that full-length blots/gels are presented in Supplementary Fig. 6. (**b**) Estimation of the Aβ amount associated with 1 μg isolated mitochondria from APP23 mice by comparison with increasing amounts of Aβ(1–40) (**c**) *in-vitro* FRANK assay of APP23 and WT mitochondria (1 μg total protein). Where indicated WT mitochondria were spiked with 1 ng of monomeric Aβ(1–40) and either tested immediately or preincubated for 30 minutes at 37 °C. Monomeric Aβ(1–40) without mitochondria was used as control. Overview of the negative staining of (**d**) membrane vesicles from P10,000 × g fraction 6 and of (**e**) mitochondria immunoisolated from cortex of depositing APP23 mice after co-immunogold labeling for Aβ peptides with monoclonal JRF/AbN/25 antibody (against the free amino terminus and the first seven amino acids of human Aβ(1–40/42) peptides)(N25) and for mitochondria using a Tom20 antibody. Two examples of each preparation show the double-labeled membrane particles adsorbed on EM grids without sectioning. The boxed region indicates the area shown at higher magnification underneath. Note the double immunogold labeling on the membrane surface for Aβ peptides (N25, 6 nm gold, black arrowheads) and Tom20 (12 nm gold, quadruple-headed arrowheads). Bars: 100 nm. Note that full-length blots/gels are presented in Supplementary Fig. 7.

To characterize the membranes within the seeding-active fractions, established markers for different intracellular membrane systems were used. A broad distribution of vesicles derived from the ER/endosomal/lysosomal system (Calnexin, Sec22b, Lamp2, Rab11) as well as exosomes, originating from the fusion of MVBs with the plasma membrane (Alix, CD63) were observed across both gradients with a peak in fractions 5/6. In contrast, markers of mitochondria and mitochondria-associated ER membranes (MAMs; Tom20, Mitofusin-2, FACL-4) were almost exclusively in fractions 5/6 of P10,000 × g (Fig. 1g). Aβ labeled membranes showed a similar distribution as they were also detected in fractions 5/6 of P10,000 × g but not P135,000 × g, which rather contained short Aβ fibrils (Supplementary Fig. S4). Interestingly, fractions 5/6 of P10,000 × g revealed a similar or higher seeding activity compared to fractions 5/6 of P135,000 × g although less Aβ was present (Fig. 1d,f). In addition, we analyzed the P10,000 × g fraction 5/6 by immunoelectron microscopy. To reduce non-specific staining associated with post-embedding immunolabelling, the isolated fractions were directly absorbed on EM-grids and double labeled for Aβ peptides and Tom20, as described for studies of exosomes^13^,^14^. Since no sectioning was performed, the membrane bilayers as well as inner mitochondrial membrane folded into cristae are not distinguishable. To avoid detection of full-length APP, a monoclonal antibody (JRF/AbN/25 (N25)^20^), which requires β-cleavage at residue 1 of Aβ was used^20^. Our immune-electron microscopy analysis revealed fragments of mitochondria and associated membranes that were strongly double labeled for Aβ and Tom20 (Fig. 2d). Remarkably, Aβ peptides did not appear as large fibrillary material on the vesicle surface. Given the Aβ and Tom20 co-labeling of membrane vesicles in P10,000 × g fraction 5/6 as observed by immunoelectron-microscopy (Fig. 2d) and the co-precipitation of Aβ and Tom20 (Supplementary Fig. S3), Aβ at mitochondria/MAMs may be a highly seeding active.

### Immuno-isolated mitochondria/MAMs from transgenic APP23 mice exhibit Aβ seeding activity *in vitro*

To independently test our hypothesis, we immunoisolated mitochondria/MAMs from WT and aged amyloid-laden APP23 cortex using a Tom22 antibody pre-coupled to magnetic microbeads. Results revealed highly purified mitochondria/MAMs that did not contain detectable amounts of alpha-tubulin, Sec22, calnexin and other non-mitochondrial makers (Fig. 2a). In contrast, Aβ and fatty acid CoA ligase 4 (FACL4; a specific marker of MAMs) co-isolated with the mitochondria (Fig. 2a), consistent with Aβ generation in MAMs^21^. Immunopurified mitochondria/MAMs were also analyzed after absorption on EM-grids as described above, and revealed double labeling for both, Aβ and Tom20 (Fig. 2e). Since Aβ associated with these membranes did not appear as large fibrillar material (Fig. 2e), it appears likely that monomeric or small oligomeric forms of Aβ are associated with the membranes. Using the FRANK assay, a strong seeding activity was also observed with the purified mitochondria/MAM preparation from APP23 transgenic but not WT animals (Fig. 2c). To investigate whether an artificial association of monomeric or small oligomeric Aβ with membranes during purification could yield similar results, mitochondria/MAMs from WT animals were mixed (either directly before analysis or with a 30-minute preincubation at 37 °C) with the same amount of synthetic Aβ as present in the APP23 mitochondria preparation (Fig. 2b,c). No increased seeding activity was noted under these conditions (Fig. 2c). Although we cannot completely exclude different association characteristics of endogenous small Aβ species, this result suggests that membrane association of Aβ is required for the seeding activity.

### Membrane fractions rich in mitochondria/MAMs and isolated mitochondria/MAMs show Aβ seeding activity *in vivo*

**Figure 3:**
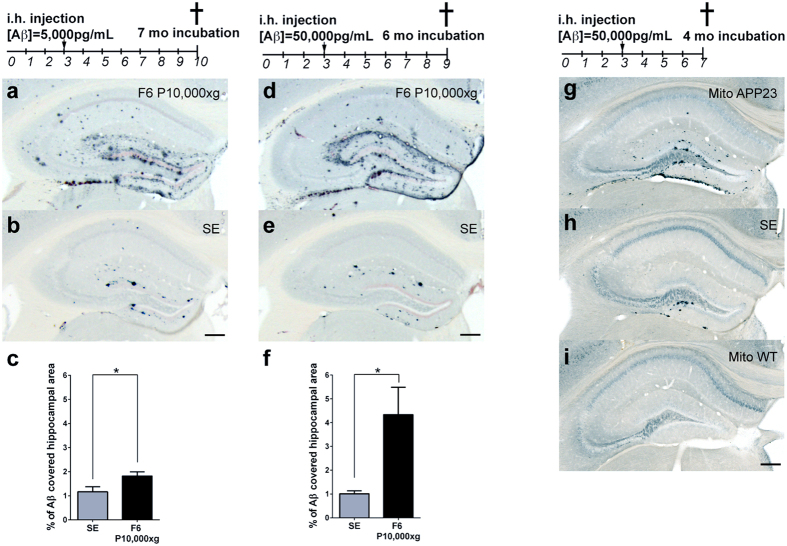
Mitochondria and associated ER membranes reveal potent β-amyloid inducing activity *in vivo*. (**a–f**) Bilateral intra-hippocampal (i.h.) injection of 3-month-old APP23 mice (n = 4–5/group) with 3000 × g supernatant (pool from 5 APP23 brain extracts; SE) or with P10,000 × g fraction 6 (pool of 3 independent preparations from 3 APP23 brains) described in Fig. 1. (**a–c**) The Aβ concentration in fraction 6 had been adjusted to 5,000 pg/ml. Brains were analyzed 7 months after injection. (**d–f**) In a second experiment mice were injected with SE (n = 5) or fraction 6 (n = 4) adjusted to a 10-fold higher Aβ concentration (50,000 pg/mL) and brains were analyzed already 6 months after injection. (**g–i**) Three-month-old APP23 mice (n = 5/group) were injected with a mix of 3 independent Tom22-immunoisolated mitochondria preparations from APP23 brain (Aβ concentration 50,000 pg/mL) and correspondingly diluted WT brains and analyzed already 4 months post-inoculation. (**a,b,d,e**) Aβ staining combined with Congo red shows amyloid deposits in hippocampus of fraction 6 (**a,d**) as well as in SE injected brains (**b,e**) Scale bar, 200 μm. (**c,f**) Quantification of the percentage of Aβ-immunostained hippocampal area. Seeding activity in P10,000 × g fraction 6 was enriched compared to SE ((**c**); *p < 0.05; unpaired t test, two-tailed, p = 0.0416, t = 2.424, df = 8) ((**f**): *p < 0.05; unpaired t test, two-tailed, p = 0.0136, t = 3.273, df = 7). (**g,h,i**) Histological analysis of Aβ deposits after brain injection with APP23 mitochondria (n = 3) (**g**) or SE (n = 5) (**h**) or WT mitochondria (n = 5) (**i**) Scale bar, 200 μm.

To confirm the seeding activities predicted by the FRANK assay *in vivo*, young APP23 mice were inoculated either with fraction 6 of the P10,000 × g gradient (Fig. 3a–f) or with mitochondria/MAMs immunoisolated from the amyloid-laden APP23 mice (Fig. 3g–i). For comparison, unfractionated brain homogenate and mitochondria/MAMs immunoisolated from age-matched WT mice were inoculated. Analysis 7 months post injection revealed amyloid induction in the host mice with fraction 6 that was 1.5-fold stronger than with the unfractionated homogenate (Fig. 3c). To obtain stronger amyloid induction that in turn facilitates quantification, the experiment was repeated with less diluted samples containing ten-fold more total Aβ (Fig. 3d–f). Despite a one-month-reduced incubation time, a 4.3-fold increase (p = 0.0136) of β-amyloid induction was observed compared to the unfractionated homogenate (Fig. 3d–f). The induced amyloid deposition showed a neuroanatomically constrained pattern indicative of spreading as described after injection of crude extracts^22^. When the highly purified mitochondria/MAMs were inoculated, a high death rate occurred for unknown reasons and the animals had to be analyzed already 4 months post injection. Nonetheless, a robust seeding activity associated with APP23 but not with WT mitochondria/MAMs was found (Fig. 3g–i).

## Conclusion

The presented study provides *in vitro* and *in vivo* evidence for an intracellular pool of Aβ seeds located at least partly at mitochondria or associated membranes. The present results do not exclude additional Aβ seeding activities e.g. residing in the soluble fraction^3^ or other membranous compartments. Our data are not the first to suggest an association of Aβ with mitochondrial or associated membrane structures. Aβ generation in MAMs and alterations of MAMs in neurons and synapses in AD and APP transgenic mice have been previously reported^23^,^24^,^25^,^26^ and an increased association of MAM and ER compartments have been suggested as a common denominator underlying AD pathogenesis^27^. Moreover, in yeast aggregated proteins were found tightly associated with mitochondria that in turn restrict aggregate mobility^21^. Given their membranous localization within neurons, intracellular Aβ seeds are candidates to contribute to the reported spreading of Aβ lesions along neuronal pathways^28^,^29^. Intracellular membrane systems including mitochondria are in dynamic association with the microtubule network when transported^30^,^31^. This functional interaction results in a physical proximity of Tau and intracellular Aβ seeds, which therefore might also contribute to the deposition of Tau aggregates and to the induction of intracellular downstream pathology.

## Methods

### Mice

Wild-type C57BL/6 (WT) and transgenic APP23 mice^32^ were bred and maintained under pathogen-free conditions at the Hertie Institute for Clinical Brain Research. All studies were performed in accordance with German animal welfare legislation and with approval from the Ethical Commission for animal experimentation of Tübingen, Germany.

### Preparation of the tissue homogenates

Aged amyloid-depositing APP23 transgenic mice (20–23 months old) and age-matched corresponding WT mice were killed by cervical dislocation followed by decapitation. The hemispheres were immediately dissected and the cerebral cortex with olfactory bulb but without cerebellum and without brain stem was homogenized in ice-cold sucrose buffer (320 mM sucrose, 5 mM Hepes pH 7.4) supplemented with protease and phosphatase inhibitors (Pierce #88668). The cortex was homogenized in 1.5 ml sucrose buffer in siliconized glass-Teflon homogenizer using 12 up-down strokes at 1,000 rpm (IKA Eurostar digital overhead stirrer).

### Differential centrifugation of homogenates and flotation sucrose density gradient centrifugation

Homogenates were centrifuged at 4 °C for 20 min at 1,200 × g. The resulting pellet was discarded and the supernatant (S1,200 × g) was collected and centrifuged at 4 °C for 30 min at 10,000 × g. The resulting pellet (P10,000 × g) was resuspended in 1.5 ml sucrose buffer. The supernatant (S10,000 × g) was centrifuged at 4 °C for 1 hour at 135,000 × g to yield a pellet fraction (P135,000 × g) and a soluble fraction (S135,000 × g). The membrane pellets (P10,000 × g and P135,000 × g) were adjusted to 1.8 M sucrose buffer with 5 mM Hepes (pH 7.4) supplemented with protease and phosphatase inhibitors and transferred into 12 ml siliconized Ultra clear Beckmann tubes. The discontinuous sucrose gradient was then layered on top of the membrane suspension by 2 ml of 1.4 M sucrose buffer with 5 mM Hepes (pH 7.4), 2 ml 1 M sucrose buffer with 5 mM Hepes (pH 7.4), 2 ml 0.6 M sucrose buffer with 5 mM Hepes (pH 7.4) and 1.5 ml 320 mM sucrose buffer with 5 mM Hepes (pH 7.4). The centrifugation of the gradients was performed in a Beckman SW41Ti rotor for 16 hours at 285,000 × g at 4 °C. Each gradient was collected in a 1 ml fraction from the top to the bottom of the tube. The pellet of the gradient was resuspended into the last fraction.

### Western blot

All sucrose density fractions were subjected to 4–12% SDS-PAGE followed by western blotting using antibodies specific for human Aβ 1–16 (1:2000; clone 6E10, Covance), rabbit anti-CD63 (1:1000; Bs-1523R, BIOSS), mouse anti-Rab11 (1:500; clone 47, BD Bioscience), mouse anti-Flotilin-1 (1:1000; clone 18, BD Bioscience), rat anti-LAMP2 (1:500; ab13524, abcam), rabbit anti-sec22b (1:5000; Synaptic Systems), rabbit anti-Tom20 (1:500; sc-11415, Santa Cruz Biotechnology), rabbit anti-calnexin (1:3000; ab22595, abcam), rabbit anti-Mitofusin-2 (1:10000 ; Sigma), rabbit anti-FACL4 (1:300 ; AP2536b, Abgent), and a polyclonal antibody against Alix (1:10,000; (gift of R. Sadoul)). Peroxidase-conjugated secondary antibodies were purchased from Jackson Laboratories (1:40,000). The immunoblots were revealed with the SuperSignal West Dura substrate (Pierce) and the chemiluminescence was recorded with a CCD camera (Stella; Raytest) and/or exposed on autoradiographic films (Hyperfilm ECL; Amersham).

### Determination of total protein and Aβ concentrations

The protein concentrations in all fractions were determined by the BCA assay (Pierce) in 96-well plates using a Mithras LB-940 reader (Berthold Technologies). The concentrations of Aβ(1–40) and Aβ(1–42) peptides were determined by electrochemiluminescence-linked immunoassay using the 96-well multi-spot human Aβ Triplex assay (Meso Scale Discovery) according to the manufacturer’s instructions and as previously described^33^.

96-well plates prespotted with the capturing antibodies Aβx-40 and Aβx-42 were blocked [1% bovine serum albumin (BSA) in Tris-buffer] and washed three times with Tris buffer. Next the crude brain extracts and fractions thereof which had been pretreated by formic acid extraction (see below) were applied (with appropriate dilution to stay within the linear range of the assay) together SULFO-TAG 6E10 detection antibody dispersed in blocking solution and incubated on the plate for 2 hours. After washing, MSD Read Buffer T was added and the plate was read immediately on a Sector Imager 6000. Data analysis used MSD DISCOVERY WORKBENCH software 2.0. Every sample was tested in duplicate and those with coefficient of variance (CV) more than 20% were excluded from analysis. Internal refrence samples were used as a control in every plate and the results were adjusted for interplate variability. Aβ concentrations were read from the standard curves using a point-to-point fit with the software SoftMax Pro 4.0 (Molecular Devices Corp.)

A formic acid extraction was performed for all fractions analyzed to monomerize oligomeric and higher aggregated Aβ species which otherwise could not be measured in the ELISA based assay. Briefly, an aliquot of each fraction was thawed on ice and resuspended in 70% formic acid (FA) final, sonicated on ice for 35 sec and then centrifuged at 4 °C for 1 hour at 25,000 × g. The FA-soluble supernatant was collected and immediately neutralized with 19 vol (v/v) 1 M Tris base, 0.5 M Na_2_HPO_4_, 0,05% NaN_3_.

### Fibrillisation of Recombinant Aβ Nucleation Kinetic (FRANK) assay

The FRANK assay was performed essentially as previously described^34^ with some modifications^18^. Briefly, all kinetic measurements were carried out with 20 μM soluble recombinant Aβ(1–40) (generously provided by Marcus Fändrich, Ulm^35^) in 50 mM Phosphate buffer pH 7.4 and 150 mM NaCl with 20 μM ThT supplemented with protease inhibitors (Complete; Roche) and with 0.1 μg total protein amounts of different isolated fractions. This normalization of the total protein concentration was implemented in order to minimize its influence on the kinetic of the FRANK assay. To accurately measure the lag phase, the *in vitro* assay has been performed with freshly monomerized Aβ1–40. Briefly, lyophylized recombinant Aβ1–40 peptide was dissolved to a stock con- centration of 5 mM in 100% DMSO and frozen at −80 °C. Before use, this stock was then freshly diluted in DMSO 1:5 to 1 mM and sonified for 10 min in a water bath followed by 5 min centrifugation at 16,100 × g at room temperature. The supernatant was then further diluted to reach 250 μM Aβ1–40 in a 50% DMSO stock. In 96-well plates (μclear non bind plate; Greiner), eight technical replicates were measured for each sample. The plates sealed with film sheets were incubated at 37 °C for up to 72–96 hours. The fluorescence measurements were performed from bottom of the plate on a Fluostar Omega plate reader (BMG Labtech) (excitation: 440 nm, emission: 480 nm) at 30-min interval, at 37 °C, after double orbital shaking for 30 sec at 500 rpm. For each sample, five to eight replicates were averaged and the lag times were determined from fitted curves^36^ with GraphPad Prism5.

### Immunoisolation of mitochondria from mouse cortex

Mitochondria were isolated using the Mitochondria Isolation kit (Miltenyi Biotec GmbH) according to the manufacturer’s instructions. Briefly, one eighth of the cortices from old wild-type C57BL/6 and depositing transgenic APP23 mice (20 to 22-month-old males) were dissected and homogenized on ice using a glass-Teflon homogenizer in 1 mL lysis buffer supplemented with protease/phosphatase inhibitors and centrifuged at 4 °C for 10 min at 750 × g. The supernatants were diluted in 10 mL separation buffer supplemented with protease/phosphatase inhibitors, and incubated with 50 μL anti-Tom22 microbeads for 1 hour at 4 °C with gentle shaking. Mitochondria were separated using LS column in the magnetic field of QuadroMACS separation unit (Miltenyi Biotec GmbH). The purity of the mitochondria and flow through was assessed further by western-blot-loading-adjusted amounts while seeding capacity was assessed in the FRANK assay as described above.

### Co-immunoprecipitations

For each immunoprecipitation, about 6 μg IgG from crude mouse polyclonal IgG (as a control) or mouse monoclonal Beta1 (AMS Biotechnology, Abingdon, UK) were added to 50 μL Protein G-coupled beads (Dynabeads^®^ Protein G beads; Novex®) diluted in 500 μL PBS supplemented with protease/phosphatase inhibitors and incubated for 1 hour at room temperature to allow antibodies to bind to the beads via their Fc-region. For preclearing of samples, 50 μL of fractions 6 from sucrose gradient of P10,000 × g were diluted in 500 μL PBS supplemented with protease/phosphatase inhibitors and incubated with Protein G-coupled beads. The precleared fractions were diluted 1:1 with ice cold PBS lacking or containing 1% Triton X-100 and were incubated with beads-antibody complexes for 1 hour at room temperature with end-over-end rotation. The unbound material (flow through) was kept and further precipitated with methanol/chloroform method. The beads were washed three times in PBS and resuspended in 2× SDS sample buffer. Flow through and bead-bound material were analyzed by SDS-PAGE and immunoblotting as described above.

### Immunoelectron microscopy

Immunolabeling of isolated membrane vesicles (fraction number 6 from the flotation sucrose gradients of the pellets P10,000 × g and P135,000 × g) and immunoisolated mitochondria was performed as previously described^37^. The samples were processed without embedding nor sectioning as follows. Drops (2 μl) of the preparation were adsorbed on formvar-carbon-coated EM-grids and fixed with 4% formaldehyde for 5 min. After blocking free aldehyde groups with 200 mM Glycine in PBS for 10 min, and with 0.5% BSA (albumin fraction-V (pH 7.0), AppliChem, Darmstadt Germany)/0.2% gelatine Merck, Darmstadt, Germany) in PBS (PBG buffer) for 5 min, the grids were incubated with the first antibody in PBG buffer for 1 hour at room temperature. The following antibodies were used: mouse monoclonal JRF/AbN/25 (N25) directed against the first seven amino acids of human Aβ(1–40/42) peptides^20^, rabbit anti-CD63/MLA1 (#bs-1523R, Bioss), rabbit anti-Alix (kindly provided by Rémy Sadoul) and rabbit anti-Tom20. After 3 washes in PBG buffer, the grids were incubated with anti-mouse IgG coupled 6 nm gold particles and anti-rabbit IgG coupled 12 nm gold particles in PBG buffer for 1 hour. After washing the grids in PBG and PBS and finally distilled water, the samples were postfixed with 1% glutaraldehyde for 5 min and then coated with 1.6% methylcellulose/0.3% uranyl acetate in distilled water.

### Intrahippocampal injection of purified fractions into transgenic APP23 hosts

Surgical procedure was performed after anesthesia with a mixture of ketamine/xylaxine as previously described^2^,^3^. Briefly, five 3-month-old APP23 mice per group received bilateral stereotactic injections (AP −2.5 mm, L ± 2.0 mm, DV −1.8 mm) of 2.5 μL samples. For each sucrose gradient fraction a mix pool from 3 independent purifications from 3 transgenic APP23 mice was prepared. The concentration of total Aβ (sum of Aβ(1–40) and Aβ(1–42) estimated by ELISA (see above) in the mix pool was adjusted with PBS to a final concentration of 5,000 pg/mL or 50,000 pg/mL. As positive control, a mix pool of 3,000 × g supernatant extracts from 5 APP23 mice (30 months old) that was diluted in PBS to a final concentration of 5,000 pg/mL or 50,000 pg/mL total Aβ was injected. The injected brains were analyzed after 6 or 7 months of incubation. For the Tom22-immunoisolated mitochondria from APP23 brain and from WT brains, a mix pool from 3 independent isolations was prepared. The Aβ concentration in the mix pool was adjusted to 50,000 pg/mL. These inoculated brains were analyzed already 4 months post injection.

### Immunohistochemistry and image acquisition

The intrahippocampally-injected mice were perfused with 4% paraformaldehyde in PBS, and the brains removed and fixed with 4%PFA overnight at 4 °C. After cryoprotection with 30% sucrose in PBS for an additional 2 days, 30 μm-tick serial coronal sections of the brains were cut using the Microm HM400 microtome. Sections were collected and stored in PBS containing 25% glycerin, 30% ethylenglycol at 4 °C until further processing. The Aβ deposits were revealed by combined staining, with Congo red dye and with a polyclonal antibody against aa1-16 of Aβ (NT-12, Novartis 1:2000). Immunostainings were performed, according to previously described standard protocol, by incubation of free floating sections with the primary antibody overnight at 4 °C, followed by incubation of the biotinylated secondary goat anti-rabbit antibody (VECTASTAIN^®^ Elite ABC system, Vector) for 45 min at room temperature. Images were acquired in brightfield mode on a Zeiss Axio Zoom.V16 microscope using a 1x/0.25NA objective and a Zeiss Axiocam HRc Rev.3.

### Quantification of amyloid plaque load

The image analysis was performed on sections through the injected hippocampus with FiJi software. Every second section starting from the frontal to the entorhinal cortex was manually delineated in Image J, and Rényi entropy measure^38^ was used to automatically determine the threshold. Percentage of covered area was directly calculated by the Fiji plugin, as a ratio between the selected hippocampus area and the thresholded structures. Statistical analysis (One-way ANOVA Bonferroni Multiple Comparisons Test) was done using GraphPad Prism 5.

## Additional Information

**How to cite this article**: Marzesco, A.-M. *et al*. Highly potent intracellular membrane-associated Aβ seeds. *Sci. Rep*. **6**, 28125; doi: 10.1038/srep28125 (2016).

## Supplementary Material

Supplementary Information
