# Supplementary material for: Highly potent intracellular membrane-associated Aβ seeds
Source: Sci Rep. 2016 Jun 17;6:28125. doi: 10.1038/srep28125 (PMC4911570; doi:10.1038/srep28125)
Supplement: Supplementary Information [file srep28125-s1.pdf]

Highly potent intracellular membrane-associated Aβ seeds

Anne-Marie Marzesco[1,2,#], Matthias Flötenmeyer[3], Anika Bühler[1,2], Ulrike Obermüller[1,2], Matthias Staufenbiel[1,2], Mathias Jucker[1,2,*] & Frank Baumann[1,2,*]

[1]Department of Cellular Neurology, Hertie Institute for Clinical Brain Research, University of Tübingen, D-72076 Tübingen, Germany; [2]German Center for Neurodegenerative Diseases (DZNE), D-72076 Tübingen, Germany; [3]Max Planck Institute for Developmental Biology, D-72076 Tübingen, Germany

Supplementary information

Supplementary Figure 1: Scheme for membrane fractions purification from mouse cortex

Supplementary Figure 2: Reduced lag time of Aβ fibrillisation in the presence of synthetic Aβ seeds. (a) The fibril formation of Aβ(1-40) peptides was followed using thioflavin T fluorescence which reveals a lag phase, followed by an increase in ThT fluorescence, after which time the maximum level of fluorescence was reached. This assay measures active Aβ seeds as they shorten the lag time until Aβ(1-40) monomers fibrilize. The FRANK assay was performed with 25 µM Aβ(1-40) in presence of either 1ng prefibrillized Aβ(1-40) or monomeric Aβ(1-40) or monomeric Aβ(1-40) dissolved in PBS and preincubated at 37°C for 30 minutes. (b) Raw data were fitted and the lag times of fibrillation of 25µM soluble Aβ(1–40) are shown in the left Y axis. Data are presented as mean ± SEM.

Supplementary Figure 3: Co-immunoprecipitation of Tom20 with β-amyloid peptides. Fraction 6 of P10,000xg membranes were subjected to immunoprecipitation using either unspecific mouse IgG, as negative control, or monoclonal antibody directed against Aβ (aa 3-6). The immunoprecipitation was performed in absence (-) or in presence of 0.5% Triton X-100. The immunoprecipitates (Bound) and unbound material (Unbound) were analyzed by immunoblotting for Aβ peptide (monoclonal Aβ 6E10) and Tom20. Fraction 6 (F6) was loaded as input and positive control.

Supplementary Figure 4: Association of β-amyloid peptides to Tom20-containing membranes

Negative staining of membrane vesicles from the fraction number 6 of the P10,000xg pellet (a-c) and of the P135,000xg (d-f) sucrose gradients after co-immunogold labeling for Aβ(1-40/42) peptides with the monoclonal JRF/AbN/25 antibody (against the free amino terminus and the seven first amino acids of human Aβ(1-40/42) peptides) (a-f)(N25, 6 nm gold, Black arrowheads) with a mitochondria marker Tom20 (a,d) (12nm gold, quadruple-headed arrowheads) or with two exosomal markers Alix (b,e)(12nm gold, asterisks) and CD63 (c,f)(12nm gold, white arrowheads). (a,b) Two examples of distinct vesicles types labeled for Aβ peptide and for Alix or CD63. (a,c) Note the two examples of heavily Aβ-labeled membrane particles enriched for Tom20. (d,e,f) Note the small Aβ fibrils present between and/or in near vicinity of small membrane vesicles in the fraction 6 of the P135,000xg sucrose gradients. Bars: 100nm

Supplementary Figure 5: Aβ fibril formation in presence of membrane fraction rich in mitochondria/MAMs

Negative staining of fibrils, obtained at the end of the FRANK assay in presence of the fraction number 6 of the P10,000xg pellet sucrose gradients, after immunogold labeling for Aβ peptides (6nm gold). Bars: 200nm (a,b) Two examples of heavily Aβ-labeled fibrils. Note the electron dense core (white dashed circle) present in the center of the fibrils aggregates, reminiscent of membranes found in the fraction 6 of the P10,000xg sucrose gradients.

Supplementary Figure 6

Full-length gels and blots for Figure 1g. Note that the blue box indicates the cropped area shown in Figure 1g. The dashed line indicates when the membrane of a blot was cut in two, and each half probed with a different antibody.

Supplementary Figure 7

Full-length gels and blots for Figure 2. Note that the blue box indicates the cropped area shown in Figure 2a-b. The dashed line indicates when the membrane of a blot was cut in two, and each half probed with a different antibody.

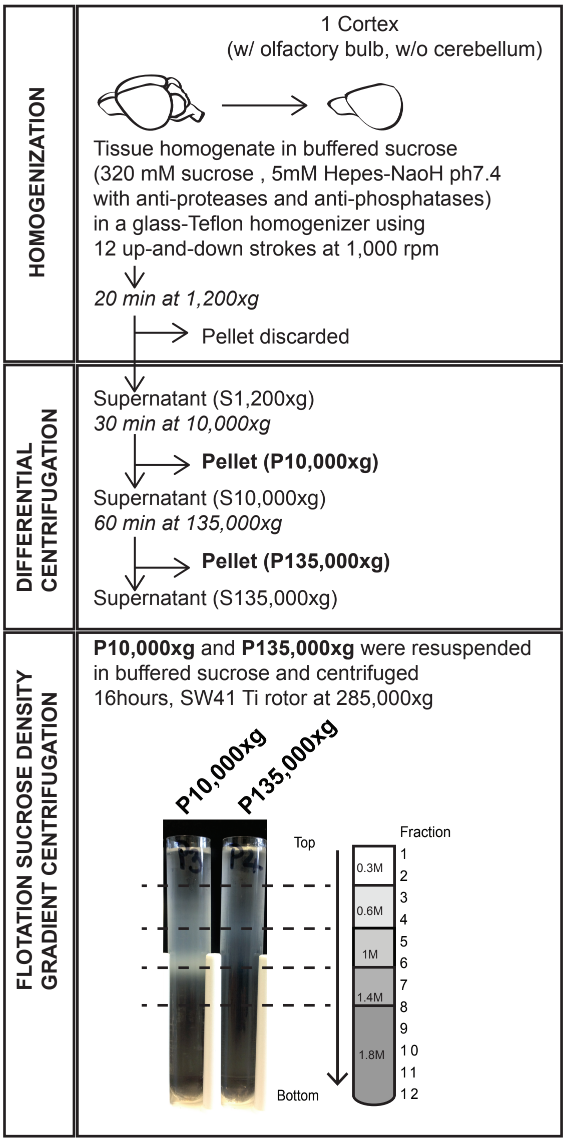
HOMOGENIZATION
1 Cortex
(w/ olfactory bulb, w/o cerebellum)
Tissue homogenate in buffered sucrose
(320 mM sucrose , 5mM Hepes-NaoH ph7.4
with anti-proteases and anti-phosphatases)
in a glass-Teflon homogenizer using
12 up-and-down strokes at 1,000 rpm
20 min at 1,200xg
Pellet discarded
DIFFERENTIAL CENTRIFUGATION
Supernatant (S1,200xg)
30 min at 10,000xg
Pellet (P10,000xg)
Supernatant (S10,000xg)
60 min at 135,000xg
Pellet (P135,000xg)
Supernatant (S135,000xg)
FLOTATION SUCROSE DENSITY GRADIENT CENTRIFUGATION
P10,000xg and P135,000xg were resuspended
in buffered sucrose and centrifuged
16hours, SW41 Ti rotor at 285,000xg
P10,000xg
P135,000xg
Top
Bottom
Fraction
0.3M
0.6M
1M
1.4M
1.8M
1
2
3
4
5
6
7
8
9
10
11
12

a

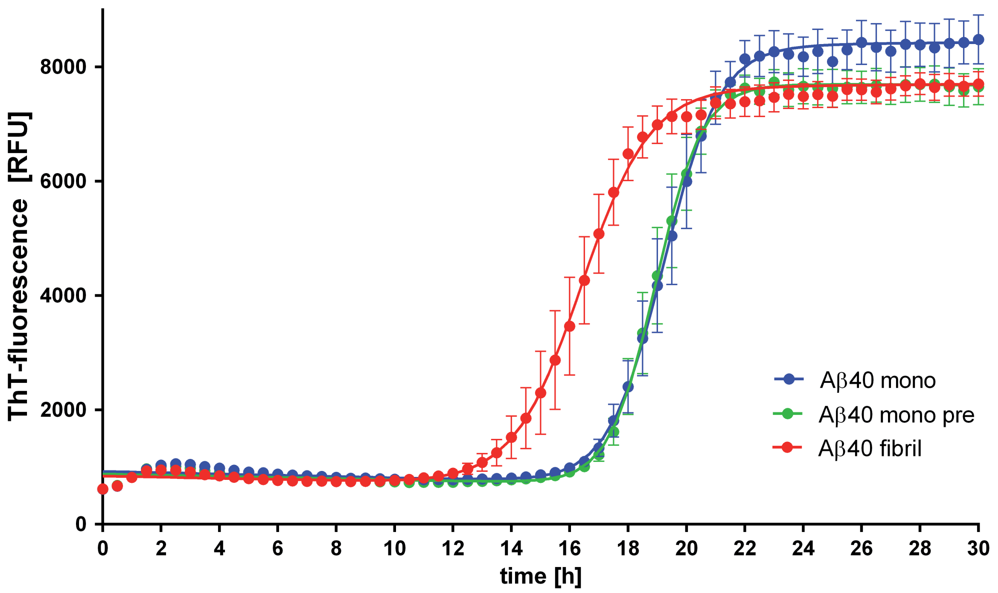
ThT-fluorescence [RFU]
8000
6000
4000
2000
0
0
2
4
6
8
10
12
14
16
18
20
22
24
26
28
30
time [h]
Aβ40 mono
Aβ40 mono pre
Aβ40 fibril

b

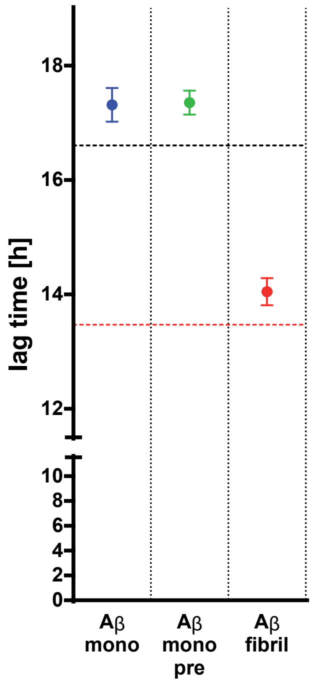
lag time [h]
18
16
14
12
10
8
6
4
2
0
Aβ mono
Aβ mono pre
Aβ fibril

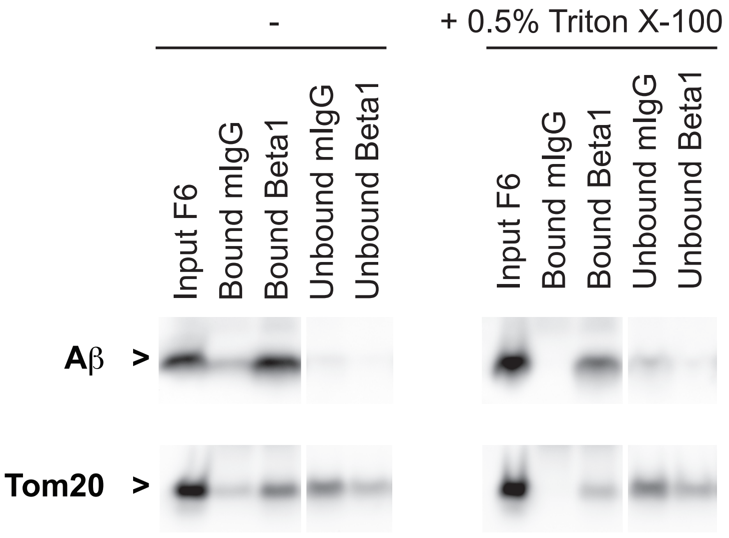

-
+ 0.5% Triton X-100
Input F6
Bound mIgG
Bound Beta1
Unbound mIgG
Unbound Beta1
Input F6
Bound mIgG
Bound Beta1
Unbound mIgG
Unbound Beta1
Aβ
Tom20

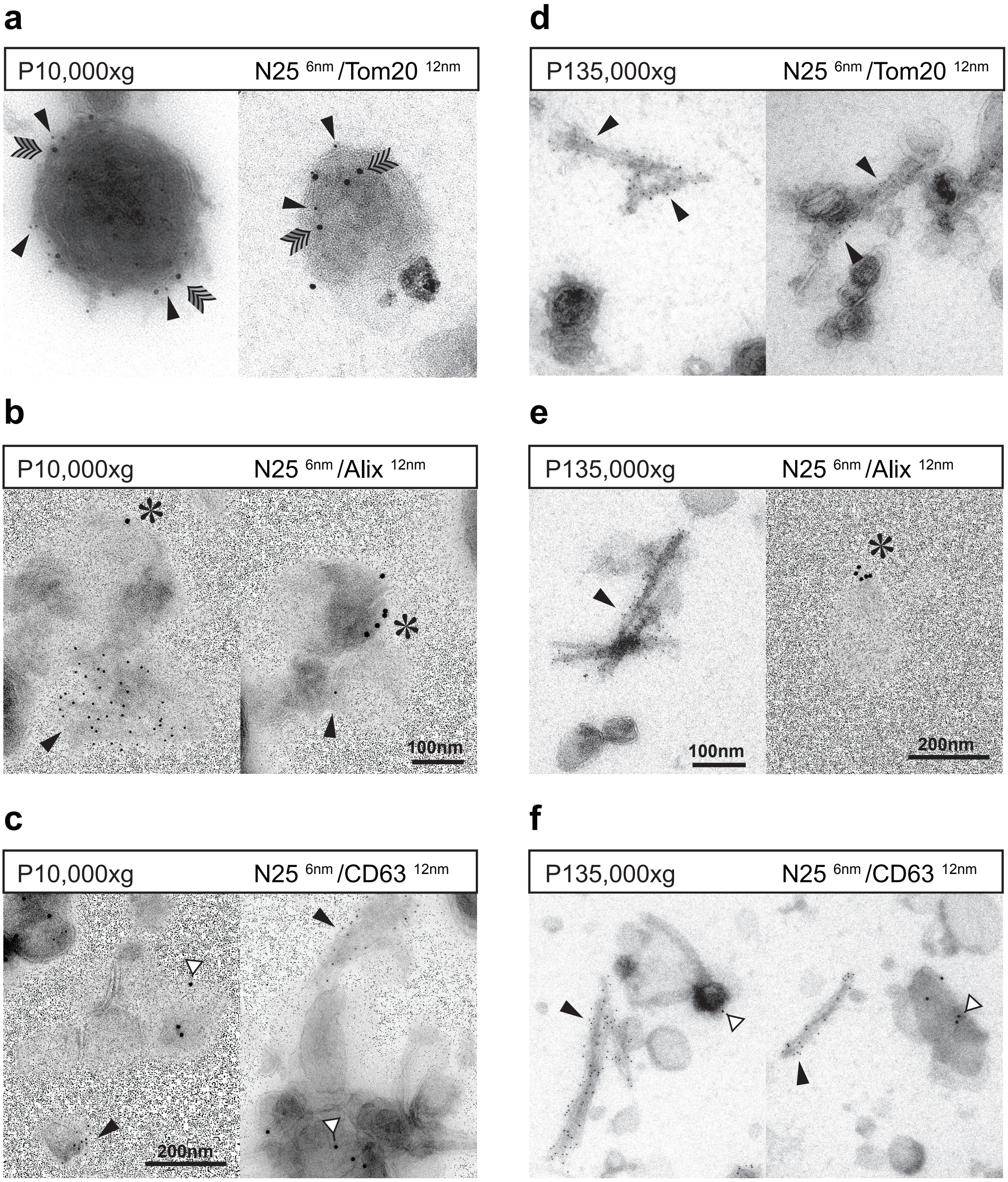
a
P10,000xg
N25 6nm /Tom20 12nm
b
P10,000xg
N25 6nm /Alix 12nm
100nm
c
P10,000xg
N25 6nm /CD63 12nm
200nm
d
P135,000xg
N25 6nm /Tom20 12nm
e
P135,000xg
N25 6nm /Alix 12nm
100nm
200nm
f
P135,000xg
N25 6nm /CD63 12nm

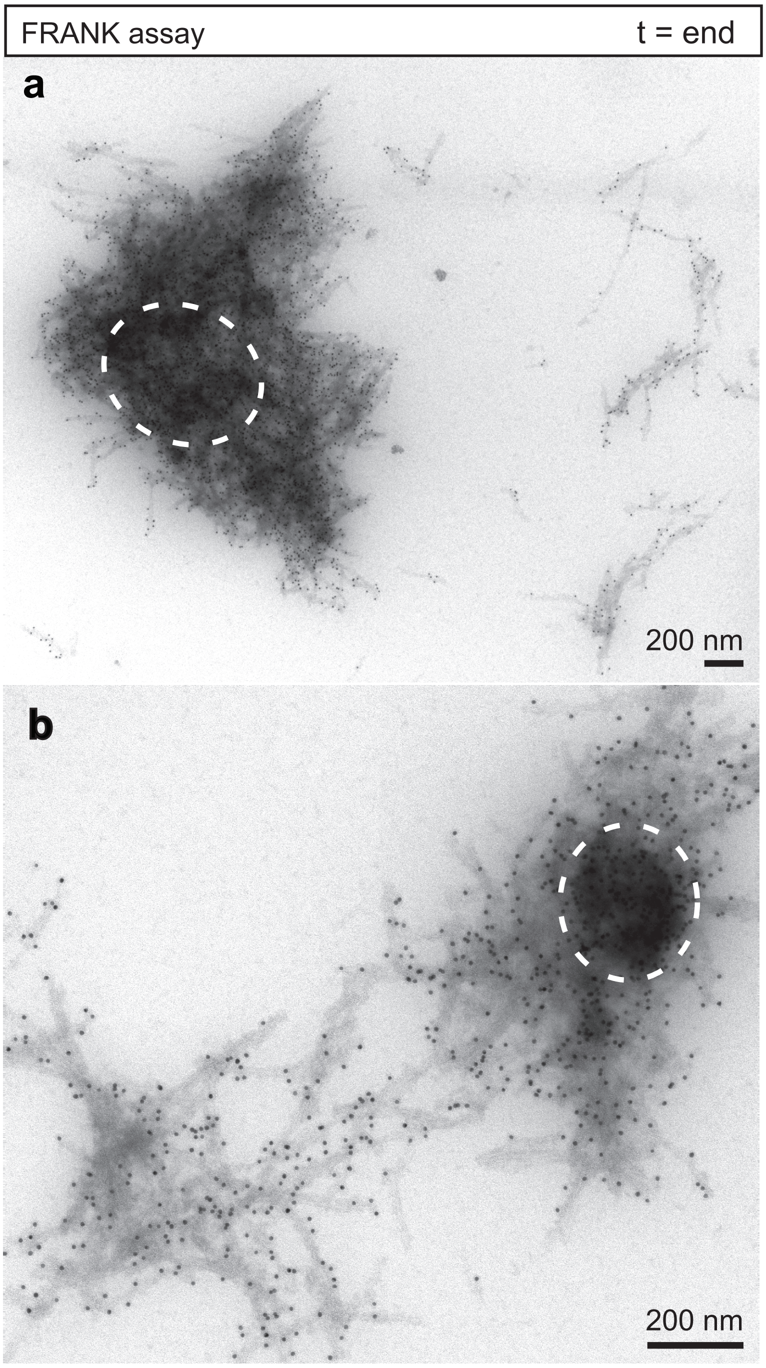
FRANK assay
t = end
a
200 nm
b
200 nm

g

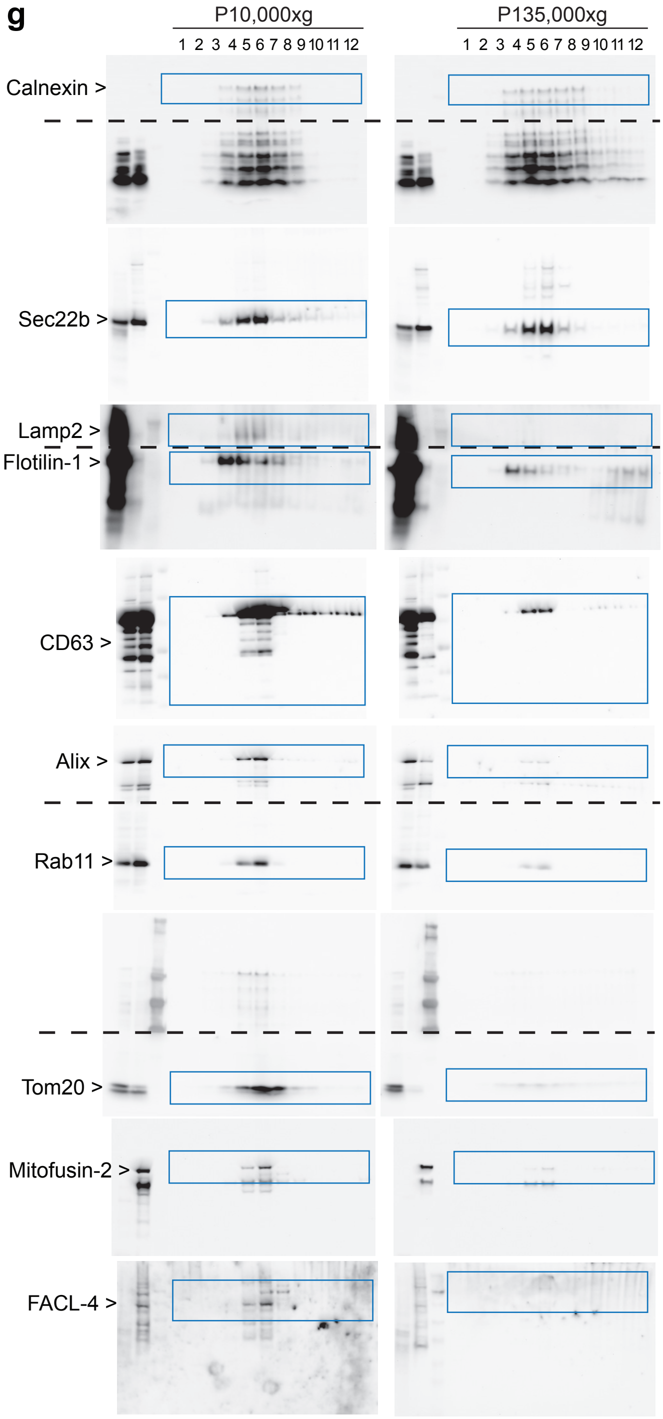

P10,000xg
P135,000xg
1 2 3 4 5 6 7 8 9 10 11 12
1 2 3 4 5 6 7 8 9 10 11 12
Calnexin >
Sec22b >
Lamp2 >
Flotilin-1 >
CD63 >
Alix >
Rab11 >
Tom20 >
Mitofusin-2 >
FACL-4 >

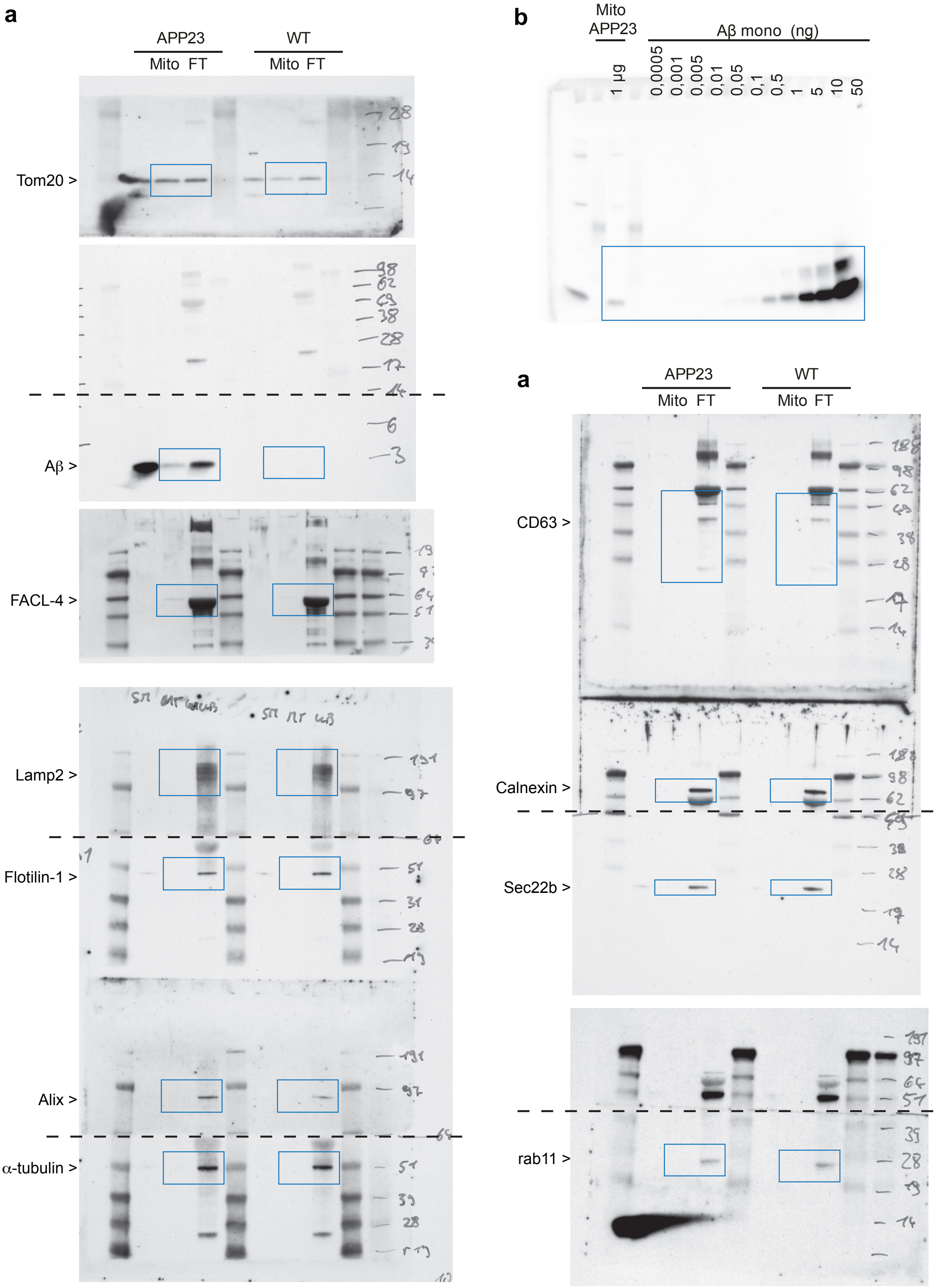

a
APP23
WT
Mito FT
Mito FT
Tom20 >
Aβ >
FACL-4 >
Lamp2 >
Flotilin-1 >
Alix >
α-tubulin >
b
Mito APP23
Aβ mono (ng)
1 µg
0,0005
0,001
0,005
0,01
0,05
0,1
0,5
1
5
10
50
a
APP23
WT
Mito FT
Mito FT
CD63 >
Calnexin >
Sec22b >
rab11 >
